# Both Nearest Neighbours and Long-term Affiliates Predict Individual Locations During Collective Movement in Wild Baboons

**DOI:** 10.1038/srep27704

**Published:** 2016-06-13

**Authors:** Damien R. Farine, Ariana Strandburg-Peshkin, Tanya Berger-Wolf, Brian Ziebart, Ivan Brugere, Jia Li, Margaret C. Crofoot

**Affiliations:** 1 Department of Anthropology, University of California Davis, 1 Shields Avenue, Davis CA, USA; 2 Smithsonian Tropical Research Institute, Balboa, Panama; 3 Edward Grey Institute of Field Ornithology, Department of Zoology, University of Oxford, South Parks Road, Oxford, UK; 4 Department of Collective Behaviour, Max Planck Institute for Ornithology, Konstanz, 78457, Germany; 5 Department of Biology, University of Konstanz, 78457, Germany; 6 Department of Ecology and Evolutionary Biology, Princeton University, 106A Guyot Hall, Princeton, NJ, USA; 7 Department of Computer Science, University of Illinois at Chicago, 851 South Morgan St, Chicago IL, USA; 8 Animal Behaviour Graduate Group, University of California Davis, 1 Shields Avenue, Davis CA, USA

## Abstract

In many animal societies, groups of individuals form stable social units that are shaped by well-delineated dominance hierarchies and a range of affiliative relationships. How do socially complex groups maintain cohesion and achieve collective movement? Using high-resolution GPS tracking of members of a wild baboon troop, we test whether collective movement in stable social groups is governed by interactions among local neighbours (commonly found in groups with largely anonymous memberships), social affiliates, and/or by individuals paying attention to global group structure. We construct candidate movement prediction models and evaluate their ability to predict the future trajectory of focal individuals. We find that baboon movements are best predicted by 4 to 6 neighbours. While these are generally individuals’ nearest neighbours, we find that baboons have distinct preferences for particular neighbours, and that these social affiliates best predict individual location at longer time scales (>10 minutes). Our results support existing theoretical and empirical studies highlighting the importance of local rules in driving collective outcomes, such as collective departures, in primates. We extend previous studies by elucidating the rules that maintain cohesion in baboons ‘on the move’, as well as the different temporal scales of social interactions that are at play.

Gregarious species—from protists to primates—all face a similar challenge: to obtain the benefits sociality provides, individuals must maintain cohesion. Theory suggests that coordinated movement can emerge from localized interaction rules between individuals and their nearby neighbours^1^,^2^,^3^,^4^, without any need for attention to or awareness of larger-scale, group level patterns of behaviour. Empirical evidence suggests that such local interaction rules underlie collective movement in a variety of types of animal aggregations, including fish schools^5^,^6^,^7^,^8^, bird flocks^9^,^10^,^11^,^12^, and insect swarms^13^,^14^. However, research to date has mainly focused on gregarious species that form large, ephemeral groups, which have few clearly differentiated inter-individual relationships, and frequently fission^15^,^16^,^17^ when faced with conflicting movement preferences. By contrast, animals that live in smaller, highly-structured societies are often familiar with all group members and can keep track of relationships among other individuals^18^. Studies of collective movement in such animal societies have largely focused on the initiation of group travel, and suggest that patterns of interaction during movement initiations are consistent with a reliance on simple, local rules, as found in larger aggregations^19^,^20^,^21^,^22^,^23^,^24^,^25^. Here we test whether interactions among nearby neighbours or social affiliates shape groups that are ‘on the move’, or whether cohesion is maintained under these conditions via generalized attention to the group as a whole.

The challenges in evaluating the dynamics that underlie coordination in moving animal groups are three-fold. First, identifying the social rules that shape group movement requires simultaneous and detailed information on the movements of all or most group members—an “observational task of daunting dimensions”^26^, especially for animals ranging in their natural habitats. Second, it is often impossible to replicate ecologically or socially relevant contexts in laboratory settings, where group tracking has typically been possible. In particular, animal groups have consistent and non-random social structure (i.e. social networks^27^,^28^,^29^,^30^,^31^) arising from long-term social processes. These long-term social dynamics can have tangible impacts on the short-term movement dynamics of animals^32^,^33^ which may not be captured in laboratory experiments, where individuals are often placed in groups with randomly selected conspecifics. Third, many potential processes can produce similar patterns, so deducing the interaction rules from observed patterns of movement presents a significant methodological challenge.

Recent advances in tracking technology have enabled the collection of high-resolution, multi-modal data on the behaviour of animal groups in the wild^34^,^35^. In this study, we fit GPS collars to 25 members of a troop of olive baboons (*Papio anubis*) at the Mpala Research Centre, Kenya^22^, representing 81% of adults and sub-adults within the troop (17 juveniles and infants were too small to collar). The collars simultaneously recorded the spatial location of each individual every second during daylight hours for 14 days, resulting in 12,684,360 synchronized observations over the study period. We study baboons because they form stable troops composed of multiple males and multiple females^18^, and have been a model system for studying the evolutionary consequences of social bonds^36^,^37^,^38^ and social structure^39^,^40^,^41^,^42^. Baboon societies are highly stratified, with clear dominance hierarchies, large variation in individual body size, and high variability in the needs and preferred foraging strategies of individuals^18^,^43^,^44^. Yet, despite these differences, groups remain highly cohesive, even while travelling large distances each day to forage on diverse and widespread foods. Studies of group departure behaviour (i.e. movement initiations) and decision-making in baboons^19^,^22^,^24^ and other primates^20^,^21^,^23^,^25^ have found evidence that is consistent with the use of local rules in initiating movement. However, the individual rules baboons use to maintain cohesion while the troop is “on the move” remain largely unknown.

The proliferation of data generated by humans, from mobile phone usage to retail rewards programs, have also led to the development of new analytical tools, notably *predictive analytics*^45^. The aim of these tools is to take observed patterns in human-generated data and use them to predict future behaviour. For example, it is well known that weather patterns can influence what shoppers buy, and thus retailers use weather forecasts to vary the stock they carry^46^. Because data are becoming more highly resolved at the individual-level, predictive analytics has increasingly been used to predict where individuals are most likely to be located. The uniqueness of human traces means that even when such data are anonymised, multiple sources of information can be linked to reconstruct individual identities (e.g. refs 47 and 48). The ability to extract more than general patterns from even coarse data has become a source of significant concerns about individual privacy^49^. However, predictive analytics, such as location prediction, also have the potential to provide insight into a range of biological processes, and could be a powerful tool for generating new hypotheses about mechanisms that underpin behaviour outside of humans.

In this study, we use predictive analytics to identify potential processes that could produce the patterns of cohesive movements observed in animal groups such as baboons. We evaluate candidate (*a priori*) models of collective movement rules by predicting the future location of individual baboons using only information about the movements of other members of their troop. Our predictive framework^50^ enables us to compare directly the performance of models based on their error (the distance between the predicted and the real location of a focal baboon at some time in the future). Models incorporate either an individual’s current neighbours or common neighbours (taken from independent samples of the data), allowing us to identify which of these sets of individuals is more predictive at different temporal scales. Further, because our models incorporate information from varying numbers of troop mates, ranging from a small number of nearest neighbours to the entire troop, we are also able to determine what neighborhood range (from local to global) provides the best predictions. By performing movement prediction, we can thus infer a likely scale at which social interactions drive group coordination.

## Results

**Figure 1:**
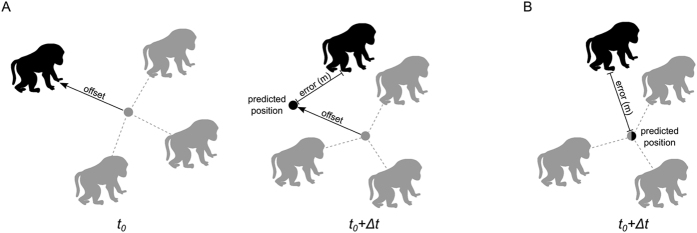
Schematic of the prediction approach. The position of the focal individual (black) at time *t*_*0*_ is calculated relative to the centroid of its neighbours. These are selected based on the rules of each candidate model (Table 1). The centroid of the location of these same neighbours is calculated at time *t*_*0*_ + Δ*t*, and the position of the focal individual is then predicted based on the current location of this centroid. For the *offset* rule (**A**), the focal individual is predicted to be located at the same position relative to this centroid as it was at time *t*_*0*_. For the *no offset* rule (**B**), the focal individual is predicted to be located at the centroid itself. The error is then defined as the distance between the focal individual’s predicted location and its observed location from the GPS data.

Each of our models predicts the future location (latitude and longitude) of a focal individual based on its position relative to a set of other group members at an initial time *t*_*0*_, and those same neighbours’ subsequent locations at time *t*_*0*_ + Δ*t*. We begin by randomly selecting 100,000 initial times in the data stream as well as a focal individual for each of these times (see Methods). We then select a subset of troop members (herein *neighbours*) based on the specifications of each candidate model (see Table 1), and calculate the focal individual’s position relative to these neighbours (Fig. 1). Based on the position of the focal individual relative to these neighbours at time *t*_*0*_, and the future locations of these same neighbours, we then predict the location of the focal individual at each time step in the future from Δ*t* = 1 up to Δ*t* = 1200 seconds (20 minutes). To avoid making predictions when individuals are simply remaining in the same location, we limit our analysis to segments of the data where the troop was moving at least at a slow pace (its centroid moved at an average speed greater than 0.1 m/sec or 120 m over the full 20-minute prediction window). We also include a model in which we select individuals in the troop at random to act as ‘neighbours’ which enables us to compare each candidate model to a null model containing the same number of neighbours but without any formal rule governing which neighbours are selected (hence generating a null hypothesis contingent on having the same number of baboons tracked). Finally, to infer the best movement rules, we statistically evaluate the relative performance of the models at performing prediction using permutation tests (see Supplementary Information).

### Which set of neighbours best predicts a baboon’s movements?

Our simplest model incorporates information about the focal individual’s position relative to its *k* nearest neighbours (herein *simple kNN*) at the initial time *t*_*0*_. We predict the location of the focal individual at *t*_*0*_ + Δ*t* using one of two basic rules:

1. *Offset:* the individual’s position relative to the centroid of its original *k* nearest neighbours (i.e. their mean position) at time *t*_*0*_ remains constant after a time lag Δ*t* (Fig. 1A).
2. *No offset:* the individual is positioned at the centroid of its original *k* nearest neighbours after a time lag Δ*t* (Fig. 1B).

**Figure 2:**
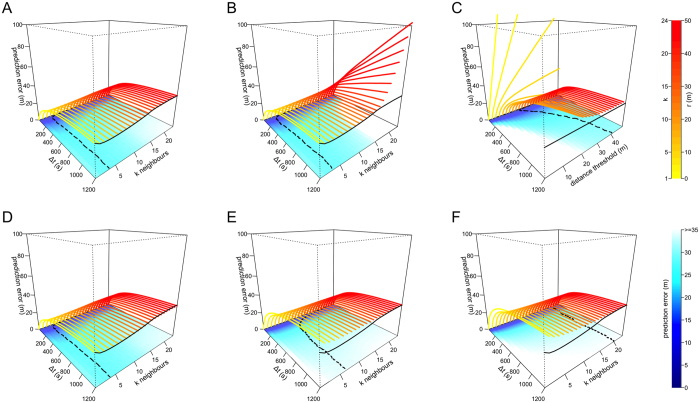
Prediction error for models based on the *offset* rule. Plots represent the following models (see Table 1): (**A**) *simple kNN*, (**B**) *density kNN*, (**C**) *metric*, (**D**) *affiliates*, (**E**) *spatial affiliates*, (**F**) *random affiliates*. Each colored line represents the prediction error in meters (z-axis) at a given time delay Δ*t* (x-axis) for a given value of *k* (panels **A,B** and **D–F**) or threshold distance *r* (**C**) on the y-axis. Solid black lines represent the prediction error for the *simple kNN* model at Δ*t* = 1200. Dashed black lines represent the *k* value with the lowest prediction error at each Δ*t* (also indicated by darker blue colors in the base of the plots).

**Figure 3:**
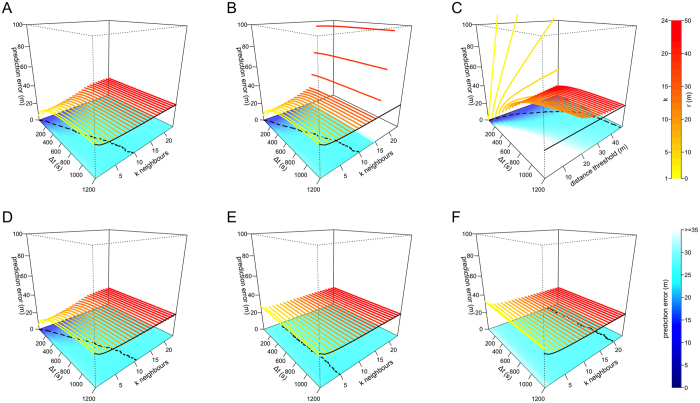
Prediction error for models based on the *no offset* rule. Plots represent the following models (see Table 1): (**A**) *simple kNN*, (**B**) *density kNN*, (**C**) *metric*, (**D**) *affiliates*, (**E**) *spatial affiliates*, (**F**) *random affiliates*. Each colored line represents the prediction error in meters (z-axis) at a given time delay Δ*t* (x-axis) for a given value of *k* (panels **A,B** and **D–F**) or threshold distance *r* (**C**) on the y-axis. Solid black lines represent the prediction error for the *simple kNN* model at Δ*t* = 1200. Dashed black lines represent the *k* value with the lowest prediction error at each Δ*t* (also indicated by darker blue colors in the base of the plots).

In both cases, the same *k* nearest neighbours from time *t*_*0*_ are used to make predictions at all subsequent times (*t*_*0*_ + Δ*t*). The *kNN* centroid is defined as the mean x-y location across the *k* neighbours. The motivation for the *no offset* model is to enable us to disentangle prediction error arising from having the wrong neighbourhood size from prediction error arising from the troop reconfiguring its relative spatial positions. The *no offset* model tests for general spatial affinity rather than exact spatial location. For location prediction over short periods of time, the *offset* model yields the smallest errors (Fig. 2A, Supplementary Figs S1–2) as the individuals do not have time to dramatically change their relative positions. At larger time scales, Δ*t* > 312 ± 40 (SD) seconds, the *no offset* model yields smaller prediction error (Fig. 3A, Supplementary Figs S3–S9). The *no offset* model outperforms the *offset* model after approximately 312 seconds, suggesting that local movement patterns lead to constant re-organisation within the moving troop over relatively short timescales.

Individuals may be attracted more strongly to clusters or dense subgroups of conspecifics than they are to those that are spread out. This could be because dense subgroups are safer from predators, or because they provide information about a good track or habitat feature. We modify the *kNN* model to incorporate variation in local density among neighbours. In this *density kNN* model, instead of calculating the centroid using the mean x-y location across the neighbours, we take the weighted mean, where the weight of each neighbor is given by 1 divided by the individual’s mean dyadic distance to the other *k*-1 neighbours. Thus, if the *k* = 5 nearest neighbours consist of 4 individuals that are very close to one another, and one that is farther away, the 5^th^ individual will have less influence on the centroid calculation (and the subsequent location prediction) in this model than it would in the standard *kNN* model. We find that incorporating the density of nearest neighbours in this way does not improve location predictions over the *simple kNN* model that does not take density into account (Figs 2B and 3B).

As an alternative to topological models (i.e. those based on a set number of nearby neighbours), some models of collective movement have assumed that individuals respond to neighbours within one or more spatially defined zones of interaction^1^,^2^,^3^. These metric models can also reproduce patterns of collective movement in many taxa, including fish^5^,^6^,^7^ and birds^11^. We therefore implement a set of *metric* models in which we predict the location of the focal individual based on the location of its neighbours within a radius *r* at time *t*_*0*_. The prediction error of *metric* models (Figs 2C and 3C) is considerably higher than models based on a fixed number of nearest neighbours. This is likely due to the substantial variation we observe in group density: the troop can expand and contract as it moves, with the mean dyadic distance typically ranging from 9.3 to 97.1 m (95% range from all *t*_*0*_ times). Thus, a neighborhood determined by a given threshold *r* sometimes contains few and sometimes contain many troop mates. Further, as the threshold distance *r* increases, the prediction error of *metric* models approach that of the global version of the nearest neighbor model because, for very large values of *r*, the metric model becomes equivalent to the *simple kNN* model where *k* = 24.

**Figure 4:**
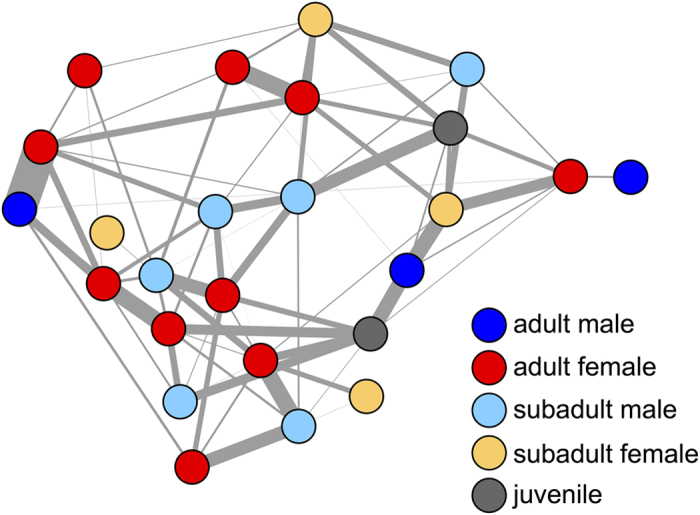
Social affiliation network based on co-sitting (a proxy for grooming). Connections (edges) between grooming partners (nodes) represent the rate of co-sitting (the number of detections in which the two individuals were stationary and observed within 1.5 meters for at least 1 minute divided by the total number of simultaneous detections of both individuals). Colors represent the ages and sexes of individuals. For visual clarity, we applied a threshold, excluding edges with weights below 0.01 from the figure.

In species with stable and well-differentiated social relationships, it is plausible that the position and movement behaviour of an individual’s close affiliates are better predictors of its movements than its dynamic neighborhood (i.e. its nearby neighbours at *t*_*0*_). In baboons, social relationships drive patterns of interaction among group members and are known to be an important determinant of individual fitness^37^,^38^. These relationships are reinforced by mutual grooming, and thus maintaining cohesion and spatial proximity with preferred social partners may be desirable, even when ‘on the move’. We test whether, given a set of neighbours *k*, individuals will maintain higher cohesion with close social affiliates than other members of the troop by weighting the centroid of the *k* nearest neighbours (from the *simple kNN* model) by their affiliation strength. In this *affiliates* model, the predicted location for the focal individual is more strongly influenced by strong affiliates than by weak affiliates. We generate an affiliation network by extracting the time that individuals spend sitting together (co-sitting, Fig. 4) as a proxy for the grooming relationships. King *et al*.^19^ found that spatial proximity and grooming were positively correlated, which suggests that co-sitting should serve as a reasonable proxy for grooming relationships. We defined edges in this network as the amount of time that a pair were observed sitting together divided by the total time both individuals were observed sitting at the same time^51^. The affiliation network is created using data from when the troop was stationary, which is completely independent (i.e. non-overlapping) from data used in the location prediction analysis, thus avoiding issues of circularity. Incorporating social affiliates yields only slight improvement in the predictive ability of models (in both the *offset* and *no offset* variants), and only when *k* is large (Figs 2D and 3D).

Although grooming plays an important role in baboon societies, a range of other relationships could lead individuals to maintain preferred spatial co-locations with a particular set of troop mates. We therefore create a general *spatial affiliation network* that describes each individual’s most common nearest neighbours^19^, drawn from 10,000 unique observations taken from when the troop is moving, but ensuring that the data do not overlap those used for prediction. For each baboon, we rank all other troop members based on the probability of being observed as the focal individual’s nearest neighbor at a particular moment given that data is available for both individuals at that time (i.e. the simple ratio index^28^,^52^). We construct a weighted and directed social network, where each edge represents this probability. We then perform the same procedure as the *simple kNN* model, but this time calculating the centroid using the focal individual’s *k* top ranked neighbours. For small values of Δ*t*, this model performs poorly (Figs 2E and 3E) relative to other models. However, as Δ*t* increases beyond 600 seconds, the *no offset* variant of the *spatial affiliates* model (Fig. 3E) performs better than any other model (including the *offset* models, Supplementary Fig. S9). This indicates that while an individual’s current neighbours are most predictive of its location at a short time scale (*simple kNN* model performs best for small Δ*t*), a set of consistent spatial affiliates tend to be more predictive of a baboon’s location over the longer term (the *spatial affiliates* model performs best for large Δ*t*). Previous studies have shown that social networks play an important role in the departure behaviour of baboons and our study suggests that the ‘follow-a-friend’ rule^19^ also plays a role in shaping their collective movement.

### How many neighbours best predict a baboon’s movement?

**Figure 5:**
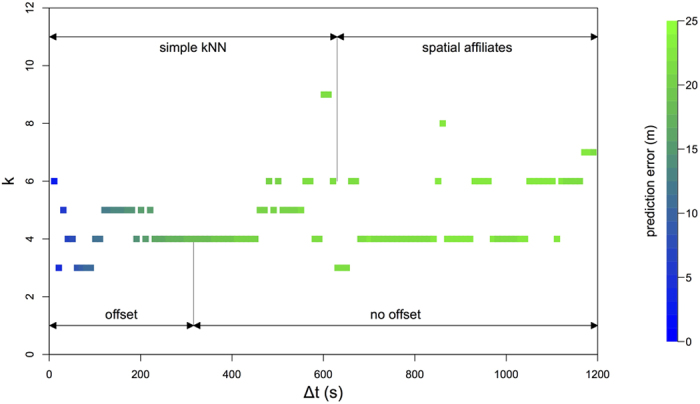
Summary of the models and parameters that best predict the location of baboons at each time delay Δ*t*. Although substantial autocorrelation results in low errors at short time delays, error rapidly stabilizes at ~20–25 m. As the prediction window increases, the best-fitting model first switches from *offset* to the *no offset* variant of the *simple kNN* model, before switching from the baseline model to the *spatial affiliates* model. This highlights the role of social affiliations in collective movement in baboons. However, across all models the best prediction accuracy is from small numbers of neighbours (4–6 neighbours).

Across all social models, predictions based on a relatively small number of nearby neighbours consistently outperform the predictions from models that include a larger number of neighbours. For medium-term predictions (30 < Δ*t* < 600), most models perform best for an optimal *k* ranging from 4 to 6 individuals. As Δ*t* becomes large (Δ*t* > 600), the most accurate prediction comes from focal individuals’ most common 4–6 nearest neighbours (i.e. the *spatial affiliates* model, see Fig. 5). To confirm that 4–6 nearest neighbours generated statistically lower prediction errors, we used a permutation procedure to test whether the difference between each pair of models was larger than expected by chance (see Supplementary Methods). We found that across a range of Δ*t* values, the model or models with 4–6 nearest neighbours were always statistically better than models with more or fewer neighbours (see Supplementary Figs S1–S8). These results suggest that when it comes to predicting the future location of a focal baboon, local models which take into account a small number of nearest neighbours provide greater accuracy than models which capture more global information about the troop.

We note that for all models, the *offset* variant consistently yields better prediction accuracy at very short timescales (Δ*t* < 30 seconds) and large values of *k*. This is because GPS error, although small, can lead to prediction errors when only few neighbours are included, but averages out across many neighbours (i.e. the centroid prediction is more precise for many neighbours). However, when comparing the error across all models and all time delays, the best predictions consistently come from models incorporating 4–6 neighbours rather than the entire group (see Fig. 5, Supplementary Figs S1–S8).

## Discussion

Theory predicts that shared decision-making should be favored in many situations^53^,^54^,^55^,^56^, and simulation models suggest that such democratic decision-making can emerge from local interactions^2^,^57^,^58^. Strandburg-Peshkin *et al*.^22^ recently demonstrated that the process of decision-making in baboons is consistent with the predictions of a theoretical model based on local movement rules^2^. Here, we use a prediction framework to demonstrate that models based on a small number of local neighbours (4–6 individuals) better capture the short-term movements of focal baboons than models based on a larger number of neighbours. Individual movements are best predicted by a relatively small neighbourhood size (compared to the size of the troop). This may partly explains why previous studies found no difference in the movement dynamics between small and large troops of baboons^24^. Whether a baboon’s current neighbours or its long-term spatial affiliates are more predictive of future location depends on the time scale in question: current neighbours are most predictive up to 10 minutes, while consistent spatial affiliates are most predictive beyond 10 minutes.

Social relationships form a substantial part of baboon life^18^. Grooming is often considered the most important currency in baboon society, enabling individuals to forge and maintain alliances, which can have significant impact on their fitness^36^,^37^,^38^. We find that incorporating information about a proxy of the grooming social network (the co-sitting network) does marginally improve location predictions at longer timescales compared to the *simple kNN* model (Figs 2E and 3E). However, we find that the most predictive model at these timescales is one that captures general spatial affiliations, rather than solely co-sitting affiliations (although we note that the co-sitting affiliation models still do better than models not including any affiliation data as Δ*t* gets beyond 800 seconds, Figs S5 and S7). This could be because a spatial proximity network is able to capture a range of different types of social relationships that combine to form group dynamics^28^,^51^. Thus, although social dynamics in baboon troops may be complex, our results suggest that a global perspective may not be necessary to shape individual movement behaviour and group coordination when the troop is ‘on the move’. Rather, baboons could be using a simple ‘follow nearby neighbours’ rule, but with some bias towards affiliates. This supports previous findings that baboons preferentially depart by following affiliates^19^, and once baboons have departed, the rule is likely to be ‘follow 4 to 6 preferred affiliates’.

That 4 to 6 individuals best predict the location of baboons ‘on the move’ fits well with what is known about their social dynamics over longer periods of time. Dunbar^59^ has argued that baboons often face constraints in the amount of time they can dedicate to social activities, and that they respond by concentrating their grooming efforts on a limited number of preferred social partners. Grooming cliques in baboons generally fit this prediction, encompassing in the range of 4–8 individuals^60^,^61^. It will be interesting for future research to investigate whom individuals follow, and how these affiliations reflect broader social relationships. Further, baboon troops are also comprised of highly heterogeneous group members, and although the aim of our study was to determine the best model for predicting locations across all baboons, individuals with different attributes, such as age, sex, or dominance could employ different variations of a general movement rule. The structure of the social network that these affiliations form could also have implications on the properties of the moving group. Theoretical work on social networks in collectively moving groups suggests that the pattern of affiliations among members could impact the structure of moving groups^33^ and their navigation accuracy^62^. Theory also suggests that individuals in central positions could have greater influence on movement directions^63^. Thus, affiliations among the members of baboon troops could play a major role in their movement, as they can do in their departures^19^,^25^.

Our results shed light on the timescales that are relevant to baboon movement decision-making and spatial organisation. We show a transition between maintaining cohesion with nearby neighbours to maintaining cohesion with a set of consistent spatial affiliates. In the human realm, this is analogous to trying to track down a friend at a party either by looking for whomever they were recently talking to or by looking for their spouse. Which of these strategies is the better choice will be determined by how long it has been since you last saw them as well as the average length of conversations. Within troops of baboons, our results suggest that the temporal scale at which the troop spatial organisation is stable during movement is only approximately 10 minutes. This result suggests that while at small time scales the most important determinant of movement in baboons is their spatially-proximate neighbours, at longer time-scales the longer-term social process of who they associate with becomes more important.

Technological advances of high-resolution animal tracking provide insight into both individual and collective movement in animal groups. However, extracting robust patterns of behaviour and evaluating hypotheses based on these types of data requires novel approaches to data analysis^35^. In this paper, we demonstrate the power of using movement predictive analytics to generate hypotheses about what rules individuals use to maintain cohesion in troops of baboons. The large number of observations available from simultaneous GPS tracking enables us to first partition the data to perform prediction, and then to evaluate the accuracy of the predictions using independent data. Constructing multiple candidate models (i.e. each of which represents a hypothesis) based on the same input data, and evaluated in exactly the same way, allows us to directly compare the accuracy of different models. It also enables us to identify the best model for each prediction window, and to extract common patterns across all models. We find agreement among models that the best predictions of baboon locations are achieved by using a small number of neighbours. We also uncover a temporal scale of group self-organization, in which random neighborhoods reshape into more consistent affiliations. Taken together, our results support theoretical work that emphasizes the role of local interactions^1^,^2^,^3^,^4^, but highlight the potential importance of long-term social relationships in shaping movement dynamics in complex societies.

## Materials and Methods

### Study site and data collection

Fieldwork was conducted at the Mpala Research Center, in central Kenya. From 21 to 29 of July 2012, we captured 33 out of the 46 members of a troop of wild olive baboons (*Papio anubis*) using individual traps (1 m^3^) baited with maize. Seven individuals were too small to fit GPS collars and were released immediately. We chemically immobilized the remainder of individuals using Ketamine (15 mg/kg). Each baboon (14 adults, 10 subadults and 2 large juveniles) was then fitted with a GPS collar (e-Obs Digital Telemetry, Gruenwald, Germany) weighing less than 5% of its body weight (D-cell battery collars = 300 g, smaller C-cell battery collars = 230 g). Collars were programmed to record location continuously at 1 Hz during daylight hours (06–18 h). Because the recording time was limited by the lifespan of smaller batteries, we report only the results of the first 14 days before the C-cell collars stopped functioning. One subadult’s collar failed almost immediately, and thus we analyzed the tracks of 25 individuals, representing 81% (23/29) of adults and subadults in the troop. Because collars recorded data continuously, positioning error was low. By conducting a test walk with pairs of collars fixed 1 m apart, we found that the average relative error between any two collars was 0.26 m (95% CI: 0.03–0.69). For full information on collar deployment and recording length, see Strandburg-Peshkin *et al*.^22^.

During the course of the study, the baboon troop ranged over an area of approximately 10 km^2^ of savannah habitat, primarily dominated by acacia scrub. Daily movements of the baboon troop consisted of group departure from a common sleep site located along a river, foraging as a cohesive group throughout the day, and subsequent return to the sleep site at the end of the day. The troop remained highly cohesive throughout each day – the upper 97.5% threshold of dyadic distances was 126 m – while traveling a median daily distance of 5 km. Their movements during the day consisted of a combination of foraging, resting, and occasional highly-directed group travel, often along roads.

### Pre-processing GPS data

Because some collars occasionally failed to log one or a few data points, we linearly interpolated these missing points based on the data before and after the missing point(s). The vast majority of the gaps in the data were only a single point (occasionally GPS collared reverted to 0.5Hz), and every 513 seconds there was a 2 second gap. A few gaps up to 30 seconds were imputed, however these represent a very small proportion of the data. To quantify the potential error associated with this procedure, we randomly selected 5000 known points and compared their true values to those inferred from interpolation. We found that the error was lower than the GPS error (<0.2 m difference between the real observed points and the interpolated points). We also used the same linear interpolation algorithm to identify points with dramatically larger errors, and replaced those in the top 99^th^ percentile with interpolated points (these points had 1 sec displacements in excess of 3.56 m). In total across the entire data set, 7.2% of the data consists of interpolated missing values, and 0.2% of data consists of replaced erroneous values.

### Location prediction

Following Li *et al*.^50^, we constructed an algorithm to perform location prediction. Our approach involves estimating the location of a focal individual Δ*t* seconds into the future after a given observation time *t*_*0*_. Based on one of 6 candidate models (see Table 1), we identify a set of baboons (for example nearest neighbours) and calculate the position of the focal individual relative to the centroid of the selected troop mates at time *t*_*0*_. We then use the centroid (mean location) of these same troop mates at time *t*_*0*_ + Δ*t* to predict the expected location of the focal individual for each value of Δ*t* (with Δ*t* ranging from 1 to 1200 seconds into the future). The procedure predicts the location of the focal individual by placing it in the same position relative to the centroid at *t*_*0*_ + Δ*t* as it was found at time *t*_*0*_ (*no offset* models; see Fig. 1a). Note that for all future times *t*_*0*_ + Δ*t*, the same individuals (those who were selected at time *t*_*0*_) are used to compute this centroid. For example, in the case of the *simple kNN* model, the *k* nearest neighbours at time *t*_*0*_ are used to predict the location of the focal individual at all subsequent future times *t*_*0*_ + Δ*t*, rather than the individual’s current nearest neighbours (those who are its nearest neighbours at *t*_*0*_ + Δ*t*). This is because using the current nearest neighbours would implicitly include information about the focal individual’s actual location into the prediction, making the calculation circular. In the case of the metric model, occasionally a radius would contain no conspecifics, in which case we followed Li *et al*.^50^ who found that the recent trajectory was the best predictor of individuals’ future trajectories when no conspecific information is included. Finally, we record the spatial distance between the real observed location and the predicted location of the focal individual as a function of Δ*t*. We perform this procedure for 100,000 randomly selected points (a random focal individual at a random time *t*_*0*_) in our data. We used exactly the same data points to estimate the error for each candidate model. However, to reduce the potential for pseudo-replication, we do not select overlapping time points for the same individual. Because we draw inferences from the relative performances of the different models, we include a statistical testing procedure to determine whether the best-performing models are significantly better at prediction than other models (see Supplementary Information).

During a prediction interval, the relative position between focal individuals and their set of neighbours can also vary due to rotation of the group. We therefore construct an alternative set of models where the focal individual is predicted to be located at the centroid of the selected neighbours (*offset* models; see Fig. 1b). We note that although this model is imperfect (for k = 1 it would predict that two individuals should be in the same position), it is useful as it tests whether individuals remain generally cohesive with their neighbours, albeit with some stochasticity to their actual location.

The overall aim of our analysis is not to simulate group-level movement dynamics, but to generate new insights into baboon movement. In particular, we are interested in revealing which networks, and how many neighbours, are most predictive of baboon movement decisions. Being able to replicate a single individual’s trajectory is an important exercise in validating movement rules as the error associated with this implementation can be much more accurately estimated than the error from simulations where the trajectory of multiple individuals are predicted. Further, the rules tested here are intentionally simplistic, as this allows both for tractability of analysis and also potentially more general insights. Thus, the candidate models we explore are unlikely to produce rules sufficient to simulate baboon trajectories, as other factors might affect individual movement, e.g. habitat features, goal-directed travel, hetero- or conspecific animals, or inherent noise. Identifying the relevance of social versus habitat features in shaping animal movement decisions is an area warranting further research.

In all distance computations, we used the latitude and longitude positions, ignoring any potential vertical displacement. We used this approximation because of the unreliability of GPS data in determining vertical displacement. However, we also expect these data to be valid due to the fact that the baboons primarily moved along the ground, and that the landscape was typically relatively flat on the scale of the baboon troop. Moreover, any imprecision introduced by this approximation would affect all models in the same way, and thus it should not bias model comparisons.

### Ethical Statement

All procedures were subject to ethical review and were approved by the Smithsonian Tropical Research Institute (IACUC 2012.0601.2015) and were carried out in accordance with the approved guidelines set out by the National Commission for Science, Technology and Innovation of the Republic of Kenya.

## Additional Information

**How to cite this article**: Farine, D. R. *et al*. Both Nearest Neighbours and Long-term Affiliates Predict Individual Locations During Collective Movement in Wild Baboons. *Sci. Rep.* **6**, 27704; 10.1038/srep27704 (2016).

## Supplementary Material

Supplementary Information

## Figures and Tables

**Table 1:** Overview of the candidate models used for location prediction.

No.	Model	Brief description of prediction
1	*simple kNN*	Centroid of *k* nearest neighbours.
2	*density kNN*	Centroid of *k* nearest neighbours weighted by their relative density.
3	*metric*	Centroid of nearest neighbours within *r* distance of the focal.
4	*affiliates*	Centroid of *k* nearest neighbours weighted by affiliation strength (a proxy for the grooming network).
5	*spatial affiliates*	Centroid of the *k* most frequent nearest neighbours.
6	*random affiliates*	Centroid of *k* randomly-selected neighbours.

Models involve calculating the centroid of a target set of troop mates and predicting the location of the focal individual using both an *offset* and a *no offset* rule (see Fig. 1). *k* values ranged from 1 to 24 (all other troop members minus the focal), and *r* values (model 4) ranged from 2 to 50 meters in increments of 2 meters.
