# Supplementary material for: Both Nearest Neighbours and Long-term Affiliates Predict Individual Locations During Collective Movement in Wild Baboons
Source: Sci Rep. 2016 Jun 13;6:27704. doi: 10.1038/srep27704 (PMC4904494; doi:10.1038/srep27704)

SUPPLEMENTAL MATERIALS

# Both Nearest Neighbors and Long-term Affiliates Predict Individual Locations During Collective Movement in Wild Baboons

Damien R. Farine[1,2,3,4,5*], Ariana Strandburg-Peshkin[6], Tanya Berger-Wolf[7], Brian Ziebart[7], Ivan Brugere[7], Jia Li[7], Margaret C. Crofoot[1,2,8*]

1. Department of Anthropology, University of California Davis, 1 Shields Avenue, Davis, CA, USA.
2. Smithsonian Tropical Research Institute, Ancon, Panama.
3. Edward Grey Institute of Field Ornithology, Department of Zoology, University of Oxford, South Parks Road, Oxford, UK.
4. Department of Collective Behaviour, Max Planck Institute for Ornithology, Konstanz, 78457, Germany.
5. Department of Biology, University of Konstanz, 78457, Germany.
6. Department of Ecology and Evolutionary Biology, Princeton University, 106A Guyot Hall, Princeton, NJ.
7. Department of Computer Science, University of Illinois at Chicago, 851 South Morgan St, Chicago IL, USA.
8. Animal Behavior Graduate Group, University of California, Davis, 1 Shields Avenue, Davis, CA, USA.

Correspondence: dfarine@orn.mpg.de (D.R.F), mccrofoot@ucdavis.edu (M.C.C.)

Supplemental methods

Testing statistical significance

To evaluate the statistical significance of our model comparisons, we employed a data permutation procedure. A model consists of three parts: the rule defining the set and weighting of neighbours, the offset or no offset, and the number of neighbours (see table 1 in the main text). The permutation test calculated the absolute mean $E^* = \overline{|E|}$ of the difference $E = E_1 - E_2$ for each prediction between a pair of models E_1 and E_2. We then created permuted datasets by shuffling predictions between the models (i.e. randomly assigning each prediction to one of two sets) and calculating the same absolute mean difference E_r^*, where *r* indicates that the difference is generated for the randomized sets. We repeated this process 1000 times for each pair of models, calculating the *P* value as the probability that the observed difference between sets of model predictions, E^*, was larger than the difference when the sets were randomized.

Each candidate model (n=288) at each time step (Δt = 1 to 1200) represents a unique model to compare to all others. This results in a very large number of P values generated for each time step (41,328). However, many of these are uninformative: for example if model A is significantly better than model B, and model B is significantly better than model C, there is likely little need to report the results of A versus C. Similarly, the results for Δt = 200 and Δt = 201 will be very similar. To reduce the number of P values to report, we first selected 4 values of Δt that represent an even coverage of Δt values (Δt = 200, 500, 800, 1200). Next, we generated a dendrogram for each value of Δt and model class (*offset*, *no offset*) to classify all the models based on their prediction accuracy. The dendrograms were generated using the neighbour-joining method implemented in the *hclust* function in R, and applied to the matrix of pairwise mean difference in prediction errors. Thus, models with a similar accuracy are plotted closer together. We then reported the P values for every branch in this tree, where a significant P value represents a case where all models on the left-hand branch are significantly different from all models on the right-hand branch.

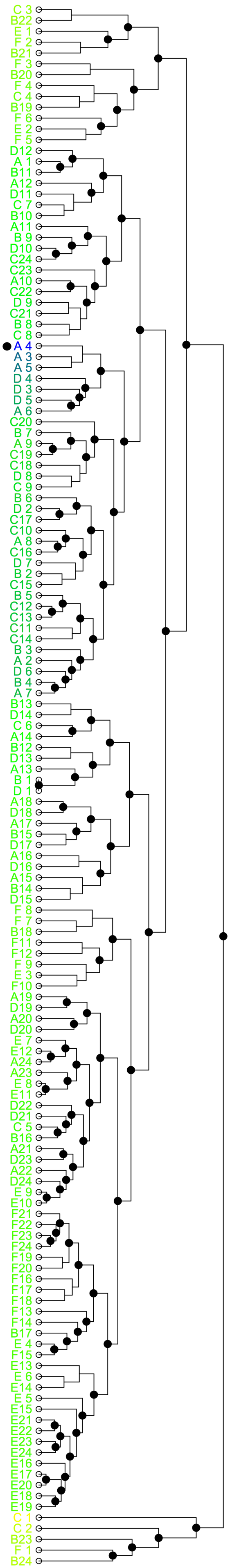

Supplemental figures

Figure S1: Dendrogram representing the difference in prediction accuracy among the candidate models for the *no offset* models at Δt = 200. Letters represent the different base models represented in panels A–F in Figures 2 and 3. The numbers represent the number of neighbours (kNN) value for the model. Black dots at a branch represent branches were all sub-models are significantly different from each other. The black dot to the left represents the model with the smallest prediction error. Colours indicate increasing prediction error (from bright blue to yellow).

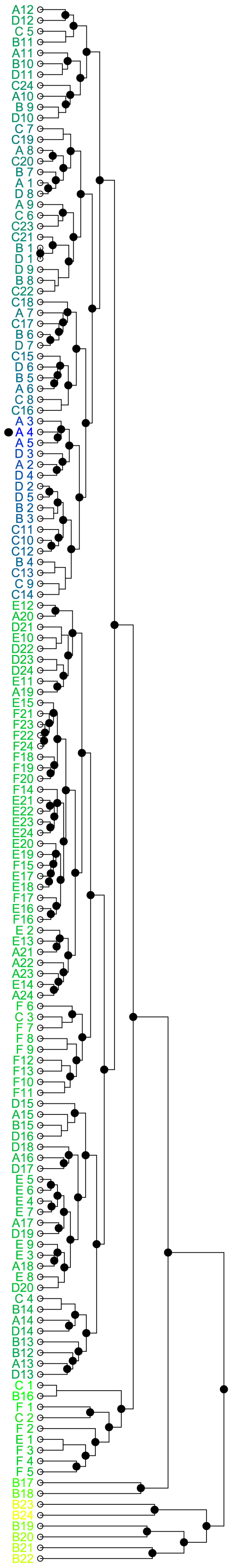

Figure S2: Dendrogram representing the difference in prediction accuracy among the candidate models for the *offset* models at Δt = 200. Letters represent the different base models represented in panels A–F in Figures 2 and 3. The numbers represent the number of neighbours (kNN) value for the model. Black dots at a branch represent branches were all sub-models are significantly different from each other. The black dot to the left represents the model with the smallest prediction error. Colours indicate increasing prediction error (from bright blue to yellow).

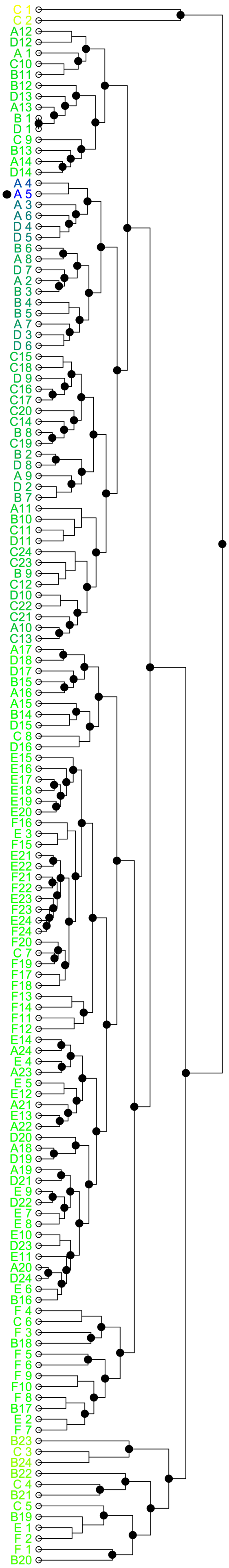

Figure S3: Dendrogram representing the difference in prediction accuracy among the candidate models for the *no offset* models at Δt = 500. Letters represent the different base models represented in panels A–F in Figures 2 and 3. The numbers represent the number of neighbours (kNN) value for the model. Black dots at a branch represent branches were all sub-models are significantly different from each other. The black dot to the left represents the model with the smallest prediction error. Colours indicate increasing prediction error (from bright blue to yellow).

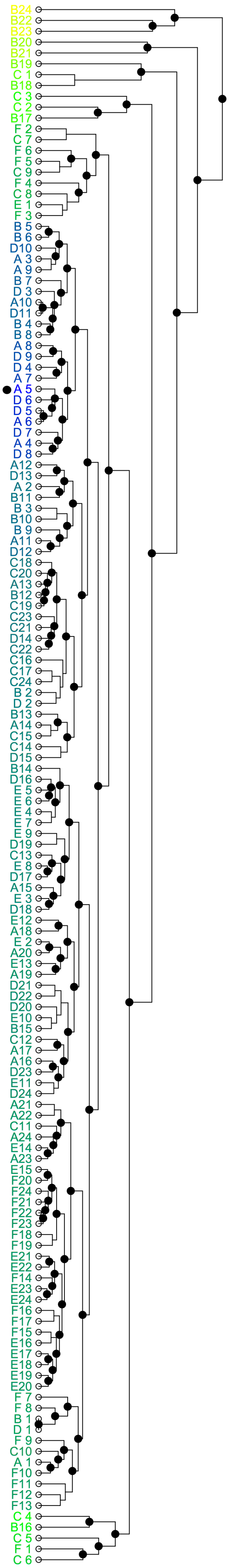

Figure S4: Dendrogram representing the difference in prediction accuracy among the candidate models for the *offset* models at Δt = 500. Letters represent the different base models represented in panels A–F in Figures 2 and 3. The numbers represent the number of neighbours (kNN) value for the model. Black dots at a branch represent branches were all sub-models are significantly different from each other. The black dot to the left represents the model with the smallest prediction error. Colours indicate increasing prediction error (from bright blue to yellow).

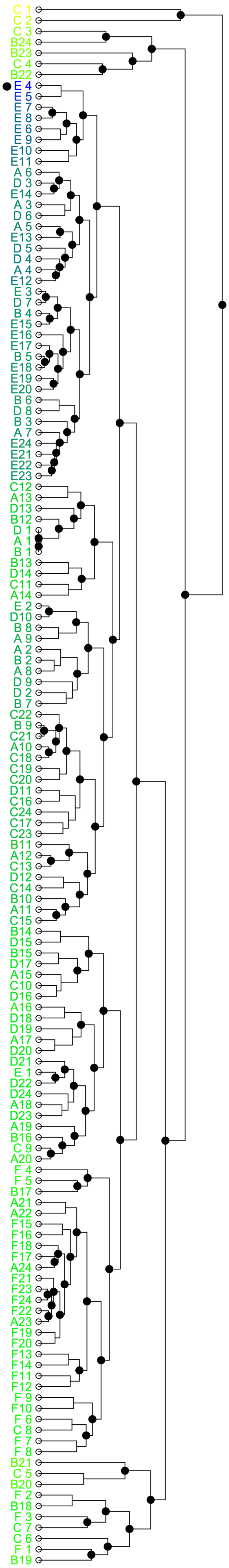

Figure S5: Dendrogram representing the difference in prediction accuracy among the candidate models for the *no offset* models at Δt = 800. Letters represent the different base models represented in panels A–F in Figures 2 and 3. The numbers represent the number of neighbours (kNN) value for the model. Black dots at a branch represent branches were all sub-models are significantly different from each other. The black dot to the left represents the model with the smallest prediction error. Colours indicate increasing prediction error (from bright blue to yellow).

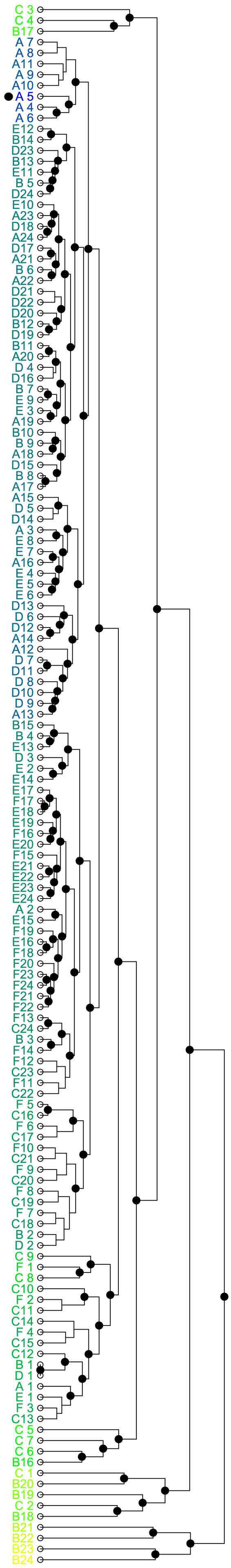

Figure S6: Dendrogram representing the difference in prediction accuracy among the candidate models for the *offset* models at Δt = 800. Letters represent the different base models represented in panels A–F in Figures 2 and 3. The numbers represent the number of neighbours (kNN) value for the model. Black dots at a branch represent branches were all sub-models are significantly different from each other. The black dot to the left represents the model with the smallest prediction error. Colours indicate increasing prediction error (from bright blue to yellow).

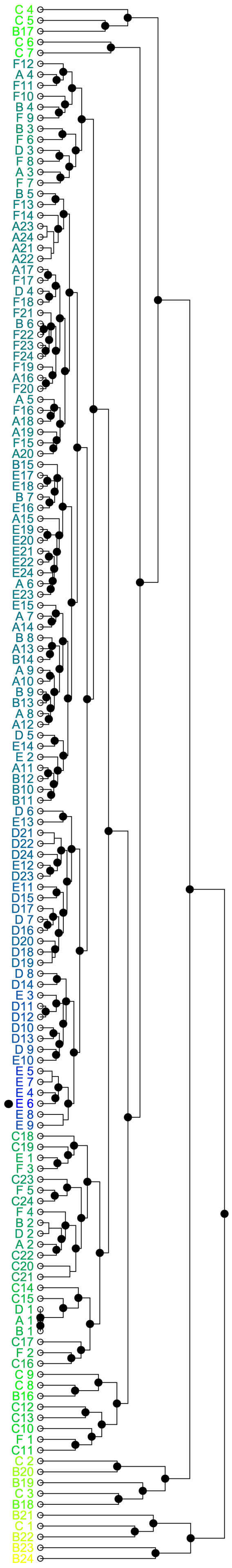

Figure S7: Dendrogram representing the difference in prediction accuracy among the candidate models for the *no offset* models at Δt = 1200. Letters represent the different base models represented in panels A–F in Figures 2 and 3. The numbers represent the number of neighbours (kNN) value for the model. Black dots at a branch represent branches were all sub-models are significantly different from each other. The black dot to the left represents the model with the smallest prediction error. Colours indicate increasing prediction error (from bright blue to yellow).

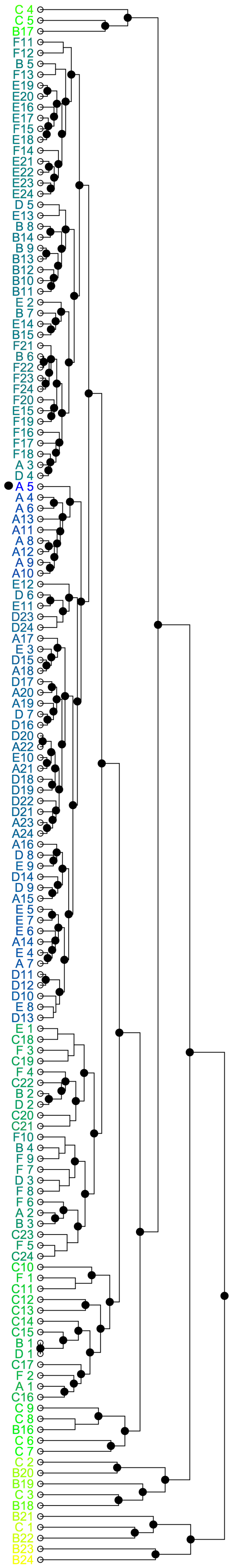

Figure S8: Dendrogram representing the difference in prediction accuracy among the candidate models for the *offset* models at Δt = 1200. Letters represent the different base models represented in panels A–F in Figures 2 and 3. The numbers represent the number of neighbours (kNN) value for the model. Black dots at a branch represent branches were all sub-models are significantly different from each other. The black dot to the left represents the model with the smallest prediction error. Colours indicate increasing prediction error (from bright blue to yellow).

Figure S9: Evaluating the statistical difference between the best *offset* and the best *no offset* models. (A) For Δt = 200, the best *offset* model had significantly lower error than the best *no offset* model. For (B) Δt = 500, (C) Δt = 800, and (D) Δt = 1200, the best *no offset* models all had significantly lower error than the best *offset* models. The differences between the model with larger error and the model with lower error are shown by the red lines. The distribution of absolute differences drawn from 1000 randomisations are shown by the blue histograms.

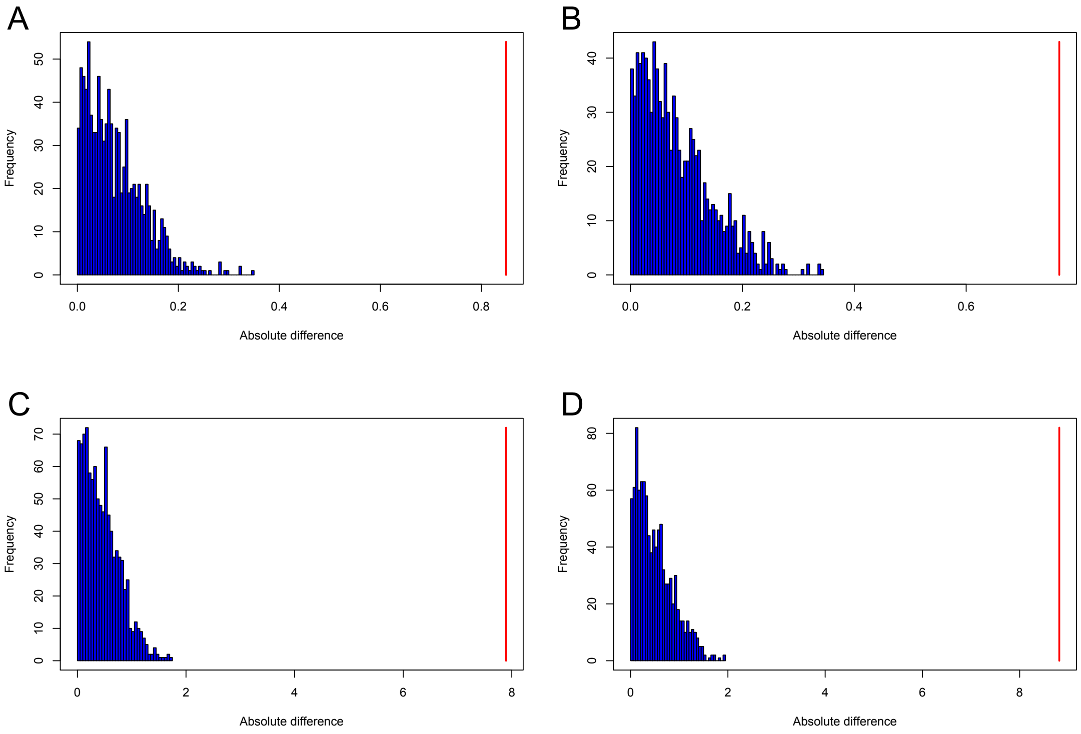

Supplement: Supplementary Information [file srep27704-s1.pdf]
